# Lumbopelvic Fixation: How to Be Less Invasive When You Cannot Be Minimally Invasive—A New Subcutaneous Supra-Fascial Approach to Minimize Open Iliac Screwing

**DOI:** 10.3390/jcm14051600

**Published:** 2025-02-27

**Authors:** Carlo Brembilla, Emanuele Stucchi, Mario De Robertis, Giorgio Cracchiolo, Ali Baram, Gabriele Capo, Zefferino Rossini, Andrea Franzini, Marco Riva, Federico Pessina, Maurizio Fornari

**Affiliations:** 1 Department of Neurosurgery, IRCCS Humanitas Research Hospital, Via Alessandro Manzoni 56, 20089 Rozzano, MI, Italy; emanuele.stucchi@humanitas.it (E.S.); mario.derobertis@humanitas.it (M.D.R.); ali.baram@humanitas.it (A.B.); gabriele.capo@humanitas.it (G.C.); zefferino.rossini@humanitas.it (Z.R.); andrea.franzini@humanitas.it (A.F.); federico.pessina@hunimed.eu (F.P.); maurizio.fornari@humanitas.it (M.F.); 2 Department of Biomedical Sciences, Humanitas University, Via Rita Levi Montalcini 4, 20090 Pieve Emanuele, MI, Italy; 3 School of Medicine and Surgery, University of Milano-Bicocca, 24127 Bergamo, BG, Italy; g.cracchiolo@campus.unimib.it

**Keywords:** lumbopelvic fixation, lumbosacral junction, iliac screw fixation, minimally invasive surgery (MIS), sacral metastasis, U-shaped cross-link

## Abstract

**Background/Objectives:** Lumbopelvic fixation (LPF) is essential for stabilizing the lumbosacral junction (LSJ) in cases of trauma, tumors, and other pathologies. While minimally invasive percutaneous techniques are preferred when feasible, open LPF remains necessary when direct sacral access is required. This study describes a modified open LPF technique designed to minimize invasiveness while maintaining effective stabilization. **Methods:** We present a case of sacral metastasis requiring LPF. The surgical technique involves a linear midline incision, meticulous subfascial dissection to preserve the Longissimus thoracis and Iliocostalis lumborum muscles, and a subcutaneous supra-fascial approach for iliac screw placement guided by intraoperative CT navigation. A U-shaped cross-link is used for final construct stability. The case illustrates the application of this technique in a 56-year-old female patient with metastatic breast carcinoma involving the sacrum, complicated by nerve compression and urinary retention. **Results:** The patient underwent successful LPF with nerve root decompression and partial tumor resection. Postoperatively, she experienced no new neurological deficits and demonstrated progressive improvement in sphincter function. The described surgical approach minimized soft tissue disruption, blood loss, and potential complications associated with more extensive dissection. Six-month follow-up CT scans confirmed the stability of the LPF construct and the residual lesion. **Conclusions:** When open LPF is unavoidable, the described subcutaneous supra-fascial approach for iliac screw placement, combined with muscle preservation and a U-shaped cross-link, offers a less invasive alternative that minimizes soft tissue trauma, reduces potential complications, and facilitates faster patient recovery. This technique can be particularly beneficial in patients with sacral metastases requiring nerve decompression and tumor resection.

## 1. Introduction

Lumbopelvic fixation (LPF) is a crucial technique for achieving solid construct stability across the lumbosacral junction (LSJ). Indications for LPF include unstable sacral fractures, pseudarthrosis, infections, and tumors at the LSJ, particularly when associated with significant bone loss and/or neurological deficits [1,2]. Biomechanically, LPF is considered superior to other approaches due to its ability to directly transfer vertical loads from the lumbar spine to the iliac bones, effectively bypassing the sacrum. Optimal placement of pelvic screws is critical for LPF success. The ideal entry point for iliac screw placement is located close to the posterior inferior iliac spine, ensuring secure fixation within the dense cortical bone of the ilium [3,4].

Traditional open iliac screw placement techniques can have several drawbacks. Extensive soft tissue dissection for accurate screw placement can increase the risk of tissue devitalization, leading to potential complications such as increased blood loss, prolonged operative times, and a heightened risk of infection [5].

Percutaneous techniques offer a less invasive alternative, allowing for rapid fixation of posterior pelvic or sacral pathologies while minimizing soft tissue disruption. They are particularly well suited for conditions requiring stabilization of the LSJ, such as many traumatic and oncological fractures [6,7,8]. However, percutaneous approaches are limited in cases requiring direct access to the sacrum for procedures such as nerve decompression, tumor resection, open reduction in sacral fractures, or sacral reconstruction [9].

This paper presents a case of sacral metastases. We describe a refined surgical technique for open LPF, developed to minimize invasiveness while ensuring robust and reliable fixation.

## 2. Case Presentation

A 56-year-old woman with a history of infiltrating mucinous breast carcinoma and ductal carcinoma in situ (DCIS) underwent right quadrantectomy in 2020, followed by adjuvant radiotherapy and oral Tamoxifen. Subsequent follow-up examinations revealed no evidence of disease recurrence.

In June 2023, she presented with left-sided gluteal pain radiating along the posterior aspect of the ipsilateral thigh. The pain was described as burning, with an initial severity of 8/10 on the Numerical Rating Scale (NRS) [10]. Further investigation with pelvic CT in November 2023 revealed an expansive lesion completely replacing the first three sacral vertebrae, with associated spinal canal invasion. Subsequent sacral spine MRI confirmed a heterogeneous, vascularized lesion measuring approximately 80 × 50 mm. Concurrently, a similar lesion was identified in the right femoral neck. A whole-body 18F-FDG PET/CT scan did not reveal any other sites of significant metabolic activity. Biopsy of the sacral lesion confirmed metastatic involvement from her previous breast carcinoma. Immunohistochemical analysis demonstrated estrogen receptor (ER) positivity, progesterone receptor (PR) negativity, and a Ki67 proliferation index of less than 3%. In January 2024, the patient underwent hypofractionated radiotherapy targeting the sacral and right femoral lesions, delivering a total dose of 46 Gy in 12 daily fractions. Concurrent systemic therapy with Ribociclib and Fulvestrant was initiated. Following radiotherapy, the patient reported a partial reduction in pain intensity to 5/10 on the NRS.

In May 2024, the patient experienced a recurrence and worsening of symptoms. Right-sided gluteal pain, now radiating to the posterior thigh, calf, and lateral foot, emerged, accompanied by tingling paresthesia. Left-sided gluteal pain persisted at a moderate intensity (NRS 8/10). Over the following weeks, she developed urinary symptoms, including urgency, incontinence, and incomplete bladder emptying.

**Figure 1:**
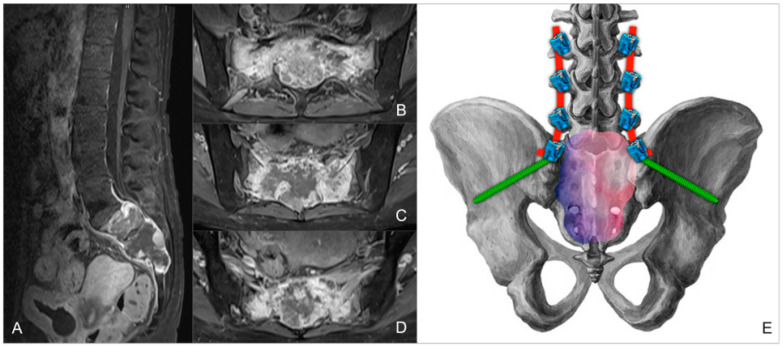
Preoperative MRI of the sacral metastatic lesion, in sagittal (**A**) and axial (**B**–**D**) planes. (**E**) Three-dimensional reconstruction of the planned LPF, including nerve root decompression and tumor resection.

In June 2024, the patient presented to the Emergency Department with acute urinary retention, necessitating the placement of an indwelling urinary catheter. Subsequent MRI (Figure 1A–D) and CT scans confirmed progression of the sacral lesion with significant compression of sacral nerve roots. Based on the clinical and radiological findings, lumbopelvic fixation (LPF) was planned, including nerve root decompression and intralesional resection of the sacral lesion (Figure 1E). The surgical procedure had a total duration of 250 min, with intraoperative blood loss of 180 cc.

The postoperative course was uneventful, with no new-onset neurological deficits and progressive restoration of sphincter function. The drains were removed on the second postoperative day, and the patient began ambulating with a lumbar elastic support. The patient was discharged home on the tenth postoperative day with a neurorehabilitation program. At discharge, she was able to perform all activities of daily living (ADL) and was free of urinary catheter, as no active urinary retention was observed at catheterization; her pain level had decreased from 8/10 to 2/10 on the NRS. Histomolecular analysis of the intraoperative tissue revealed metastatic involvement of breast carcinoma with a triple-negative phenotype (ER negative, PR negative, Ki67 1%, HER-2 low − score 1+). Given the systemic disease control achieved with the ongoing treatment despite the triple-negative profile of the lesion, therapy was modified to include Ribociclib and Fulvestrant in combination with Denosumab to manage lytic bone involvement. Six-month-follow-up CT scans demonstrated the stability of the residual lesion and the LPF construct.

## 3. Surgical Technique

**Figure 2:**
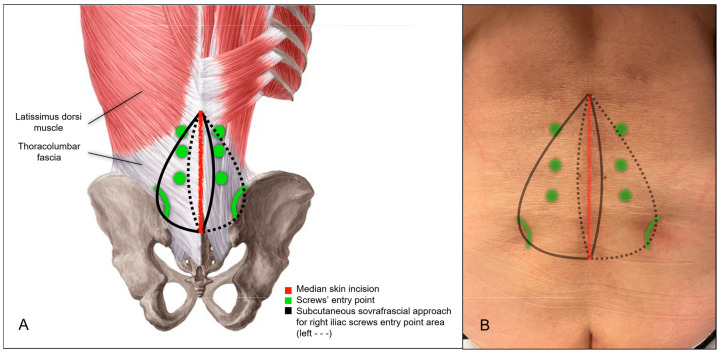
Surgical approach for LPF. (**A**) Illustration of the linear lumbosacral skin incision and the subcutaneous supra-fascial dissection to expose the PSIS. (**B**) Six-month postoperative view of the surgical field, demonstrating the well-healed incision.

**Figure 3:**
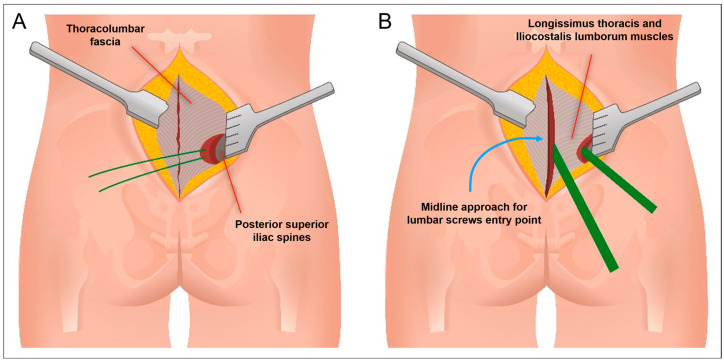
(**A**) Subcutaneous exposure of the thoracolumbar fascia and posterior superior iliac spine (PSIS). (**B**) Creation of the submuscular/subfascial tunnel via digital dissection (digitoclasia), preserving the distal attachments of the Longissimus thoracis, Iliocostalis lumborum, and Multifidus muscles.

**Figure 4:**
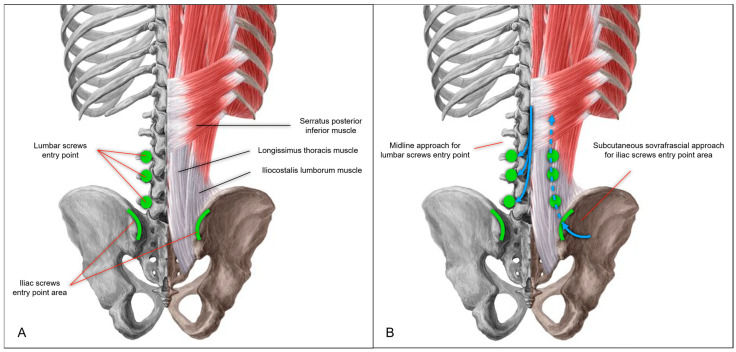
(**A**) Illustration of lumbar and iliac screw entry points. (**B**) Midline approach for lumbar screw entry points (left), and subcutaneous sovrafrascial approach (right) for iliac screw entry point area.

The patient was positioned prone. Intraoperative CT imaging was performed using a Medtronic O-arm™ Navigation System (Littleton, MA, USA). A midline longitudinal skin incision was made from L3 to the sacrum. Subcutaneous dissection exposed the supraspinous ligament between L3 and the sacrum. The thoracolumbar fascia was then dissected bilaterally, extending to the level of the posterior superior iliac spine (PSIS) (Figure 2A,B and Figure 3A). The subcutaneous dissection was extended sufficiently to allow comfortable access to the PSIS for screw placement and was wide enough to permit subsequent retraction of the skin and subcutaneous tissues for the tightening of fixation rods. Standard midline skeletonization was then performed to expose the vertebrae from L3 to the sacrum and identify the lumbar screw entry points. Medial portions of the Longissimus thoracis and Multifidus muscles were carefully disinserted to expose the vertebrae, while preserving the lateral muscle mass for optimal function and minimizing soft tissue trauma. Following frame placement for intraoperative CT navigation, polyaxial screws were inserted into the L3, L4, and L5 vertebral bodies (Figure 4A,B).

**Figure 5:**
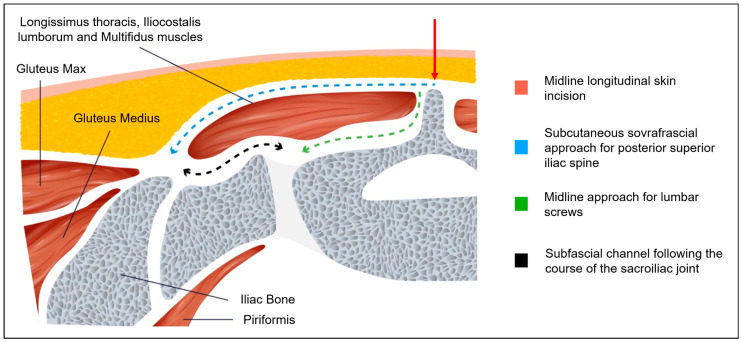
Cross-sectional view at the S1 level: (red) midline longitudinal skin incision; (blue) subcutaneous supra-fascial approach for PSIS; (green) midline approach for lumbar screws; (black) subfascial/submuscular channel for rod insertion.

For iliac screw placement, the thoracolumbar fascia was incised along the medial margin of the PSIS bilaterally. A small portion of the Iliocostalis lumborum muscle was dissected to expose the medial margin of the PSIS, creating sufficient space for iliac screw insertion. Two iliac screws were placed per side using the subcutaneous supra-fascial approach (Figure 4A,B). A submuscular/subfascial channel was then created by blunt dissection, connecting the space obtained at the level of the iliac spines to the midline surgical cavity. With their fingers (digitoclasia: digital dissection), the surgeon gently dissected along the bony plane, following the course of the sacroiliac joint (Figure 3B and Figure 5). This digital dissection technique, linking the medial and PSIS surgical fields, is crucial for maintaining the integrity of the distal attachments of the Longissimus thoracis and the majority of the Iliocostalis lumborum and the Multifidus muscles, thereby minimizing muscular trauma. Pre-contoured rods with appropriate lordosis were then inserted through the medial surgical cavity, passed beneath the previously created submuscular/subfascial tunnel, and positioned in the tulips of the iliac screws. In this case, the rods were connected to the iliac screw tulips using dedicated connector. The previous creation of adequate subcutaneous space is paramount for successful rod placement, as it allows for sufficient retraction of the superficial soft tissues and prevents compression or impingement. The rods were secondarily secured within the tulips of the lumbar screws, for easer manipulation through the median approach.

**Figure 6:**
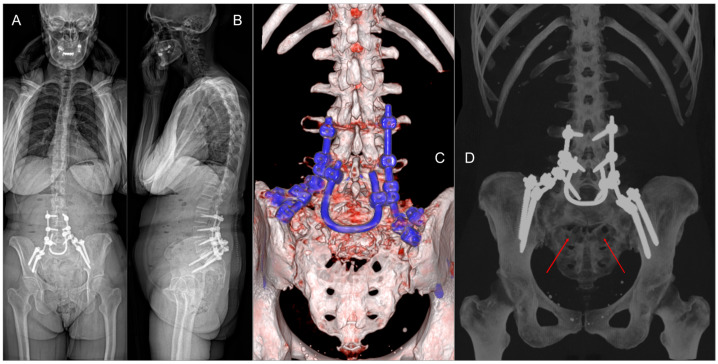
(**A**,**B**) Post-operative standing X-ray. (**C**,**D**) Six-month 3D CT scan reconstruction demonstrating the stability of the LPF and the residual sacral defect (red arrows).

A sacral laminectomy was then performed. The spinal canal was found to be occupied by the tumor, compressing the cauda equina nerve roots. Careful debulking of the sacral tumor was performed to decompress the nerve roots (subtotal resection—STR). A U-shaped cross-link, crafted from a 5.5 mm diameter rod, was finally placed between the rods through the midline approach and secured with dedicated connectors. At the end of the procedure, the fixation system demonstrated stability, and the nerve roots were adequately decompressed (Figure 6A–D). Lumbopelvic fixation was achieved using polyaxial titanium alloy screws from the Cortical Fix Expedium Spinal System (DePuy Synthes, LLC, a Johnson & Johnson Company, New Brunswick, NJ, USA).

During closure, the thoracolumbar fascia was meticulously sutured at the level of the PSIS, and the supraspinous ligament too. Two drains were placed: one in the midline subfascial surgical cavity and one in the subcutaneous layer.

## 4. Discussion

LSJ pathologies present a complex surgical challenge due to the unique anatomical and biomechanical characteristics of this transitional zone between the mobile spine and the relatively fixed pelvis. The inherent biomechanics of the LSJ, coupled with the destructive nature of pathological processes affecting the sacrum, create a significant challenge for spinal stabilization. LPF is a cornerstone of LSJ stabilization, leveraging the human pelvis as a stable anchor point. While various implants have been used over the years, iliac screw fixation has become increasingly prominent due to its demonstrated ability to prevent LPF failure and sacral fractures [3,11,12].

Open LPF is a major procedure. The extensive muscle and soft tissue dissection required for accurate iliac screw insertion can increase the risk of tissue devitalization, leading to increased blood loss, prolonged operative times, and infection. Percutaneous approaches, often guided by intraoperative CT navigation or robotic assistance, aim to mitigate these risks and allow for rapid LPF [8,12,13]. These minimally invasive techniques are limited when direct access to the sacrum is necessary for procedures such as nerve decompression, tumor resection, open reduction in sacral fractures, or sacral reconstruction.

The S2-alar-iliac (S2AI) screw has recently gained significant popularity as it provides similar biomechanical fixation without the extensive tissue dissection associated with traditional iliac screws [14]. Highly destructive lesions of the sacral spine, however, may preclude S2AI instrumentation [15].

In our experience, we have prioritized minimizing invasiveness even in open LPF procedures requiring sacral access. Linear skin incision is less problematic than U-shaped or inverted Y-shaped (Mercedes star) incisions. The sacral region is prone to pressure sores postoperatively, especially given that patients rest in the supine position. Extensive dissection can make early mobilization difficult. A single linear incision helps maintain better vascularization of the soft tissues, reducing the risk of necrosis and infection [16].

Maintaining a functionally intact muscle plane is crucial. Preserving the Longissimus thoracis and Iliocostalis lumborum muscles maintains the functionality and support of the posterior paravertebral muscle compartment. Dissection between the subcutaneous plane and the muscle fascia allows easy access to the PSIS. While the minimal detachment of the Longissimus thoracis muscle provides sufficient access for iliac screw placement, precise screw placement within this limited exposure is critically dependent on intraoperative CT navigation due to the lack of anatomical landmarks. Following screw placement, the supraperiosteal space needed for rod placement between the Longissimus muscle and the sacroiliac region can be easily created with smooth dissection.

In pathologies requiring open sacral access for partial or total sacrectomy and LSJ reconstruction, the resulting cavity from bone resection presents a challenge [9,17,18]. Preserving the Longissimus thoracis and Iliocostalis lumborum muscles helps maintain a separation between the cavity and the external surface, supporting the overlying subcutaneous tissues and skin. For final LPF construct torsional stability, the cross-link is essential. This additional stability is crucial for minimizing micromotion at the LSJ, promoting bone fusion, and reducing the risk of implant failure. The recent literature has highlighted the strength and resistance of U-shaped cross-links in LSJ constructs [19,20], among other solutions. Moreover, in our experience, the U-shaped cross-link also acts as a support for the overlying tissues (muscle, subcutaneous tissue, and skin), minimizing tissue depression above the surgical cavity.

These minimally invasive surgical techniques have, in our experience, resulted in reduced blood loss, less tissue necrosis, and lower risk of infection, facilitating faster patient mobilization and reducing the risk of pressure sores.

This surgical technique is the result of years of clinical expertise gained by our team in the treatment of traumatic sacral fractures. Starting from a traditional open approach, we have progressively developed a refined method that maintains the strength of fixation while significantly reducing invasiveness. This article presents a single clinical case, which inherently limits the generalizability of our findings and the ability to definitively assess the overall effectiveness and safety of the described technique. While the presented case demonstrates the successful application of the method in a specific clinical scenario, it cannot provide statistically significant evidence of efficacy or identify any potential complications that may arise in a larger patient population, including the risk of infection, implant loosening, and neurological complications. Specifically, this case report cannot establish the technique’s applicability to other patient populations with varying comorbidities, disease severity, or anatomical variations. Furthermore, the absence of a control group limits our ability to compare the outcomes of this technique with those of traditional open or minimally invasive approaches. Finally, the short-term follow-up period in this case report precludes the assessment of long-term outcomes, such as implant stability, pain relief durability, and the potential for late complications.

## 5. Conclusions

LPF provides excellent stability for LSJ pathologies. Percutaneous or robotic-assisted methods offer the least invasive approach when feasible. However, these techniques are not always applicable, particularly in cases of traumatic and, especially, oncological pathologies requiring sacral access. In such cases, the described subcutaneous supra-fascial approach for iliac screw placement, combined with the use of a U-shaped cross-link, represents, in our experience, a surgical technique that minimizes the major drawbacks of open LPF, including hemorrhage, tissue necrosis, prolonged recovery, pressure sores, and infection.

## Data Availability

We excluded the data availability section since our study did not report on any data present in public datasets.
